# The anti‐obesity and anti‐diabetic effects of the edible seaweed *Gloiopeltis furcata* (Postels et Ruprecht) J. Agardh in mice fed a high‐fat diet

**DOI:** 10.1002/fsn3.3100

**Published:** 2022-10-17

**Authors:** Shigeru Murakami, Chihiro Hirazawa, Toshiki Mizutani, Rina Yoshikawa, Takuma Ohya, Ning Ma, Yutaka Owaki, Toyohiro Owaki, Takashi Ito, Chiaki Matsuzaki

**Affiliations:** ^1^ Department of Bioscience and Biotechnology Fukui Prefectural University Fukui Japan; ^2^ Fukui Bioincubation Center (FBIC) Fukui Prefectural University Fukui Japan; ^3^ Division of Health Science, Graduate School of Health Science Suzuka University Suzuka Japan; ^4^ Owaki Manzo Syoten Co. Ltd. Fukui Japan; ^5^ Research Institute for Bioresources and Biotechnology Ishikawa Prefectural University Nonoichi Japan

**Keywords:** diabetes, *Gloiopeltis furcata* (Postels et Ruprecht) J. Agardh, obesity, polysaccharide, seaweed

## Abstract

Obesity and diabetes are serious, chronic medical conditions associated with a wide range of life‐threatening conditions. The aim of this study was to investigate the effects of the edible red seaweed *Gloiopeltis furcata* (Postels et Ruprecht) J. Agardh (*G. furcata*) on the development of obesity, diabetes and related metabolic diseases in mice. Male C57BL/6J mice were fed a high‐fat (HF) diet (60% energy as fat), or an HF diet containing 2% (w/w) or 6% powdered *G. furcata* for 13 weeks. Polysaccharides of *G. furcata* were isolated and their anti‐inflammatory effects were evaluated in lipopolysaccharide‐stimulated RAW264.7 cells. The HF diet group showed greater weight gain, lipid accumulation in the body and liver, and increased serum levels of glucose and cholesterol in comparison to the normal group fed a normal diet (10% energy as fat). The treatment of HF diet mice with *G. furcata* reduced these changes and stimulated the fecal excretion of fat. In addition, *G. furcata* suppressed the HF diet‐induced elevation of inflammation and oxidative stress markers in the serum and liver. The isolated sulfated polysaccharide from *G. furcata* inhibited pancreatic lipase activity and decreased the production of nitric oxide and TNF‐α in the murine macrophage cell line RAW264.7. These results show that *G. furcata* treatment can attenuate obesity, diabetes, hepatic steatosis, and dyslipidemia in mice fed an HF diet, which is associated with inhibited intestinal fat absorption and reduced inflammation and oxidative stress by a sulfated polysaccharide.

## Graphical abstract

Edible red seaweed *G. furcata* alleviates obesity and associated metabolic disorders in mice fed a high fat diet by suppressing intestinal lipid absorption, oxidative stress and inflammation.

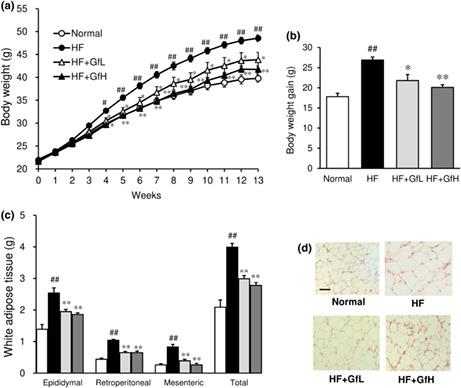

## 1 INTRODUCTION

The prevalence of overweight and obesity is increasing at an alarming rate, not only in industrialized countries but also in developing countries (Blüher, 2019). Obesity is characterized by abnormal deposition of fat in the adipose tissue, resulting from an imbalance between caloric intake and energy expenditure (Oussaada et al., 2019). Obesity is associated with a wide variety of adverse outcomes, including type 2 diabetes, insulin resistance, inflammation, cardiovascular diseases, and cancer (Maggio & Pi‐Sunyer, 2003).

Seaweed has been consumed in daily meals in Japan, Korea, and China since ancient times. Seaweed intake, as an ingredient in Japanese dishes, is considered to have contributed to the health and longevity of Japanese people. Seaweed has few calories and contains a wide variety of biologically active compounds, including soluble fibers (polysaccharides), minerals, vitamins, polyphenols, and carotenoids (Mark et al., 2017). Recent large‐scale epidemiological studies among Japanese have revealed that dietary seaweed intake is inversely associated with the risk of cardiovascular mortality (Kishida et al., 2020; Murai et al., 2019). The growing importance of daily diet in the prevention of obesity and related diseases results in the need for natural nutritional products. Based on its nutritional advantage, seaweed has drawn increased attention as a healthy ingredient in recent years (Brown et al., 2014).

*Gloiopeltis furcata* (Postels et Ruprecht) J. Agardh (*G. furcata*), also known as *funori* in Japan, is a red seaweed that grows on the upper part of the rocky intertidal zone in countries on the North Pacific coast, including Japan and Korea. It contains a high‐molecular sulfated polysaccharide called funoran (Ren et al., 1994), which is used for adhesives, food additives, cosmetics, shampoos, and the restoration of paintings in Japan, because of its viscosity and moisturizing properties. *G. furcata* has also long been applied as a folk medicine in Japan and China from old times. The health‐promoting effects of *G. furcata* are described in old Japanese and Chinese medicine and pharmacy books. *Yamato Honzo* is an ancient document on biology and agriculture published by Japanese medical scientists in 1709 describing the effects of various types of foods such as vegetables, seaweed, and herbal medicine. Among them, *G. furcata* is described as a food material having a health‐promoting effect. *The Compendium of Materia Medica* or *Bencao Gangmu* is a comprehensive medical book published in China during the Ming dynasty of the 16th century also describing the beneficial effects of *G. furcate*.

Several studies have described the biological and pharmacological effects of *G. furcata*. Extracts of *G. furcata* and isolated sulfated polysaccharides exhibit antioxidant, anti‐inflammatory, and antitumor activities (Shao et al., 2013; Yang et al., 2010). The methanol extract from *G. furcate* improves exercise performance in mice (Sutikno et al., 2019). It also shows anti‐cancer activities in the human hepatocarcinoma HepG2 cells (Bae & Choi, 2007). We have previously reported that *G. furcata* contains high concentrations of taurine, a sulfated amino acid derivative that plays an important role as an organic osmolyte in the body (Kawasaki et al., 2017).

Given its high content of sulfated polysaccharides and polyphenols, *G. furcata* is expected to have beneficial effects on metabolic dysfunction, including obesity and diabetes. In fact, oligosaccharides from *G. furcata* have been shown to improve the metabolism of lipids and glucose in HepG2 cells (Wang et al., 2020). However, the effects of *G. furcata* on metabolic disease have not been determined in animal models. In the present study, we investigated the effect of chronic treatment with *G. furcata* on the development of obesity, diabetes, and related metabolic disorders in mice fed a high‐fat (HF) diet.

## 2 MATERIALS AND METHODS

### 2.1 Preparation of powder and extracts of *G. furcata*

The *G. furcata* sample was harvested on the coast of Nagasaki Prefecture and washed with water. It was dried, reduced to a fine powder using a food mixer, and mixed into the HF diet. For the enzyme inhibition experiments, powdered *G. furcata* was extracted with 70% ethanol. Ethanol extract was prepared by homogenizing the sample powder in 70% ethanol and left at room temperature for 2 days. The sample was centrifuged at 3000 *g* for 15 min. The supernatant was then dried by evaporation and used for the lipase inhibition assay.

### 2.2 Purification and molecular mass analysis of polysaccharide

Extraction and purification of polysaccharide from *G. furcata* was carried out via the previously reported procedure (Hu et al., 2012). In brief, the powdered 100 mg of *G. furcata* was extracted with 10 ml of 85% ethanol at 80°C for 4 h (three times) to remove lipids. Polysaccharide in the residue was extracted with 10 ml of cold water, dialyzed against distilled water, and then freeze‐dried (13.0 mg). The total sugar content was determined by the phenol‐sulfuric acid method using galactose as a standard (DuBois et al., 1956). Sulfate content was determined by the BaCl_2_‐gelatin method (Ji et al., 2013) using κ‐carrageenan (TCI, Tokyo, Japan) as a positive control. The protein content was determined using a BCA protein assay kit (Pierce, Rockford, IL, USA). The relative molecular weight of polysaccharide was determined by high‐performance size exclusion chromatography with Shodex OHpak SB‐807G (Guard), SB‐807 HQ, and SB‐806M HQ (8.0 mm ID × 300 mm length; Showa Denko KK, Tokyo, Japan) at 40°C and estimated using dextran standards (150, 270, and 670 kDa from Sigma Aldrich Corp., 3755 kDa from American Polymer Standards Corp., Mentor, OH, USA). Samples (injected volume: 20 μl) were eluted using 0.3 M NaNO_3_ at a flow rate of 1 ml/min and were detected using a refractive index (RI) detector RID10 (Shimadzu Corp., Kyoto, Japan).

### 2.3 Animal experiments and dietary treatment

Six‐week‐old male C57BL/6J mice were purchased from CLEA Japan Inc. (Tokyo, Japan) and housed in a controlled atmosphere (22 ± 1°C at 50% relative humidity) with a 12‐h light/dark cycle. After 1 week of acclimation, the animals were randomly divided into four groups of 12–13 animals, as follows: (1) normal diet (Normal) group, high‐fat (HF) diet group, group with HF diet supplemented with 2% *G. furcata* (HF+GfL), and a group with HF diet supplemented with 6% *G. furcata* (HF+GfH). The compositions of the experimental diets were adjusted by considering the nutritional components of *G. furcata* as described previously (Murakami et al., 2021). The normal diet provided 354 kcal/100 g of energy (14.4% calories from protein, 11.1% calories from fat and 74.5% calories from carbohydrate), while the HF diet provided 493 kcal/100 g of energy (17.9% calories from protein, 60.7% calories from fat and 21.4% calories from carbohydrate). All experimental diets were based on the AIN‐76 diet (Oriental Yeast Co. Ltd., Tokyo, Japan). Animals were allowed free access to diets and drinking water. The diets, including HF diet, were exchanged with new ones every day. The body weight and food intake were monitored twice a week. After 13 weeks of feeding, the mice were deprived food overnight, and blood samples were withdrawn under mixed anesthetic agent (0.3 mg/kg of medetomidine, 4.0 mg/kg of midazolam, and 5.0 mg/kg of butorphanol; Fujifilm Wako Pure Chemical Co., Osaka, Japan). Liver tissue and epididymal, peritoneal, and mesenteric white adipose tissues were removed, weighed, and stored at −80°C. Some of the mice were used for histological examinations. All experimental protocols were approved by the Institutional Animal Care and Use Committee of Fukui Prefectural University (Approval No. 19‐14).

### 2.4 Serum biochemical analyses

Serum was obtained by centrifugation at 1500 *g* for 15 min at 4°C. The serum levels of total cholesterol, high‐density lipoprotein (HDL)‐cholesterol, triglyceride, alanine aminotransferase (ALT) and aspartate aminotransferase (AST) were analyzed using a Hitachi 7060 Automatic Analyzer (Hitachi, Tokyo, Japan) with commercial kits (Fujifilm Wako Pure Chemical Co., Osaka, Japan). The non‐HDL cholesterol levels were calculated by subtracting the HDL‐cholesterol from the total cholesterol level. The serum insulin (Morinaga Institute of Biological Science, Yokohama, Japan), adiponectin (Otsuka Pharmaceutical Co. Ltd., Tokyo, Japan), interleukin‐6 (IL‐6) (Fujifilm Wako Pure Chemical Co., Osaka, Japan), and tumor necrosis factor‐α (TNF‐α; Fujifilm Wako Pure Chemical Co., Osaka, Japan) levels were also determined using a commercial ELISA kit. Serum malondialdehyde (MDA) levels were determined using a commercial kit (Japan Institute for the Control of Aging Co. Ltd., Shizuoka, Japan). The homeostasis model assessment insulin resistance (HOMA‐IR), an insulin resistance index, was calculated using the following equation: HOMA‐IR = fasting glucose (mg/dl) × fasting insulin (ng/ml)/22.5.

### 2.5 Liver analyses

Lipids were extracted from the liver according to a previously described method (Murakami et al., 1997). In brief, the frozen liver tissues (50 mg) were homogenized in five volumes of isopropanol. The homogenate was kept at room temperature for 2 days and then centrifuged at 1000 *g* for 10 min. Aliquots of the supernatant were analyzed for triglyceride content using a commercial kit (Fujifilm Wako Pure Chemical Co., Osaka, Japan). Liver samples for biochemical analysis were homogenized in five volumes of cold 20 mM Tris–HCl buffer (pH 7.4), and centrifuged at 12,000 *g* for 15 min at 4°C. The lipid peroxidation was determined by estimating malondialdehyde (MDA) using a commercial kit (Japan Institute for the Control of Aging Co. Ltd., Shizuoka, Japan). The inflammatory cytokine levels in the liver were quantified using enzyme‐linked immunosorbent assay (ELISA) kits specific for mouse TNF‐α and IL‐6 (Fujifilm Wako Pure Chemical Co., Osaka, Japan).

### 2.6 Histological analyses

The mice were anesthetized with an intraperitoneal injection of mixed anesthetic agent (0.3 mg/kg of medetomidine, 4.0 mg/kg of midazolam, and 5.0 mg/kg of butorphanol; Fujifilm Wako Pure Chemical Co.), and transcardially perfused with a fixative containing 4% paraformaldehyde and 1.5% glutaraldehyde in phosphate‐buffered saline (PBS). After perfusion, the liver and white adipose tissue were removed and allowed to stand in the same fixative for 1 day. The tissues were rinsed several times with PBS and embedded in paraffin. Tissues were cut into 5‐μm‐thick sections, mounted on slides, and stained with hematoxylin and eosin (H&E).

### 2.7 Glucose tolerance test

A glucose tolerance test was performed 1 week before the end of the experiment. The mice were fasted overnight and intraperitoneally injected with glucose (2 g/kg body weight). Blood samples were collected from the tail veins of the mice, and glucose levels were measured at 0, 30, 60, 90, and 120 min after injection using a blood glucometer Nipro Stat Strip (Nipro, Osaka, Japan).

### 2.8 Fecal analyses

During fecal collection, mice were separated, and fecal samples were collected for a 24‐h period from each mouse and weighed. These samples were ground into a powder in a mortar, and 50 mg of feces was extracted with 300 μl of distilled water. After centrifugation (16,000 *g*, 30 min, 4°C), ethanol was added to the supernatant (final concentration of 85%), and polysaccharides were obtained as the precipitate. The resulting residue was washed with 85% ethanol and dried. The residue was then resuspended in distilled water and centrifuged (16,000 *g*, 10 min, 4°C), and the polysaccharide content in the supernatant was measured using a phenol‐sulfuric acid method, which has been described elsewhere, with galactose as the standard (DuBois et al., 1956). For the measurement of triglycerides, lipids were extracted by adding isopropanol (10 times the weight) to the fecal powder. The sample was then dried and dissolved in isopropanol. The concentration of triglyceride was measured using a commercial kit (Fujifilm Wako Pure Chemical Co., Osaka, Japan).

### 2.9 Anti‐inflammatory effects on RAW264.7 cells

RAW264.7 cells were prepared to a concentration of 2 × 10^5^ cells/ml using MEM medium (M5650; Sigma Aldrich) supplemented with 10% bovine fetal bovine serum and antibiotics (penicillin 100 U/ml and streptomycin 100 μl/ml) The cell suspension was added to a 96‐well plate and cultured in a 5% CO_2_ incubator for 24 h. After washing each well with phosphate‐buffered saline, lipopolysaccharide (LPS; derived from *Escherichia coli* O111, Fujifilm Wako Pure Chemical Co., Osaka, Japan) with a final concentration of 100 ng/ml and polysaccharide solution were added to the medium. The cells were cultured in a 5% CO_2_ incubator for an additional 24 h. The culture supernatant (100 μl) was collected, 100 μl of Griess reagent was added, and the mixture was left for 20 min in the dark; the absorbance was then measured at 543 nm. The nitric oxide concentration in the medium was calculated from the standard curve prepared separately. Cell viability was evaluated by colorimetric quantification via the MTT method (3‐(4,5‐dimethylthiazol‐2‐yl) ‐2,5‐diphenyltetrazolium bromide).

### 2.10 Lipase assay

The inhibitory activity of *G. furcata* extracts on lipase was determined as previously described [20] (Bendicho et al., 2001), with some modification. In brief, lipase (type II, from porcine pancreas, 400 units/mg protein; Sigma‐Aldrich Corp., St. Louis, MO) was dissolved in distilled water at 5 mg/ml and then centrifuged (1000 *g*, 5 min) and the supernatant was used as the enzyme source. 4‐Nitrophenyl butyrate (4‐NPB; Sigma‐Aldrich Corp., St. Louis, MO) was dissolved in dimethyl sulfoxide. The reaction mixture contained 100 μl of enzyme solution and 100 μl of *G. furcata* extract in 4 ml of 20 mM Tris–HCl buffer pH 8.5. The mixture was pre‐incubated at 37°C for 10 min. The reaction was started by the addition of 100μl of 5 mM 4‐NPB solution and then incubated for 30 min at 37°C. The absorbance was measured at 400 nm.

### 2.11 Statistical analysis

Results are expressed as the mean ± *SEM*. Data were analyzed by a one‐way analysis of variance (ANOVA) followed by Tukey's multiple range tests. *p* Values of *p* < 0.05 were considered to indicate statistical significance.

## 3 RESULTS

### 3.1 Isolation of polysaccharide

Polysaccharide of *G. furcata* was isolated with a yield 13.0%. It showed a symmetric peak on high‐performance size‐exclusion chromatography, and the molecular mass was estimated to be 1.8 × 10^6^ Da. The polysaccharide contained 67.1% sugar and 18.9% sulfate with a small amount of protein (1.0%). The sulfate content of *G. furcata* was similar level to that of κ‐carrageenan (17.8%) used as a positive control.

### 3.2 Body weight and food intake

**FIGURE 1:**
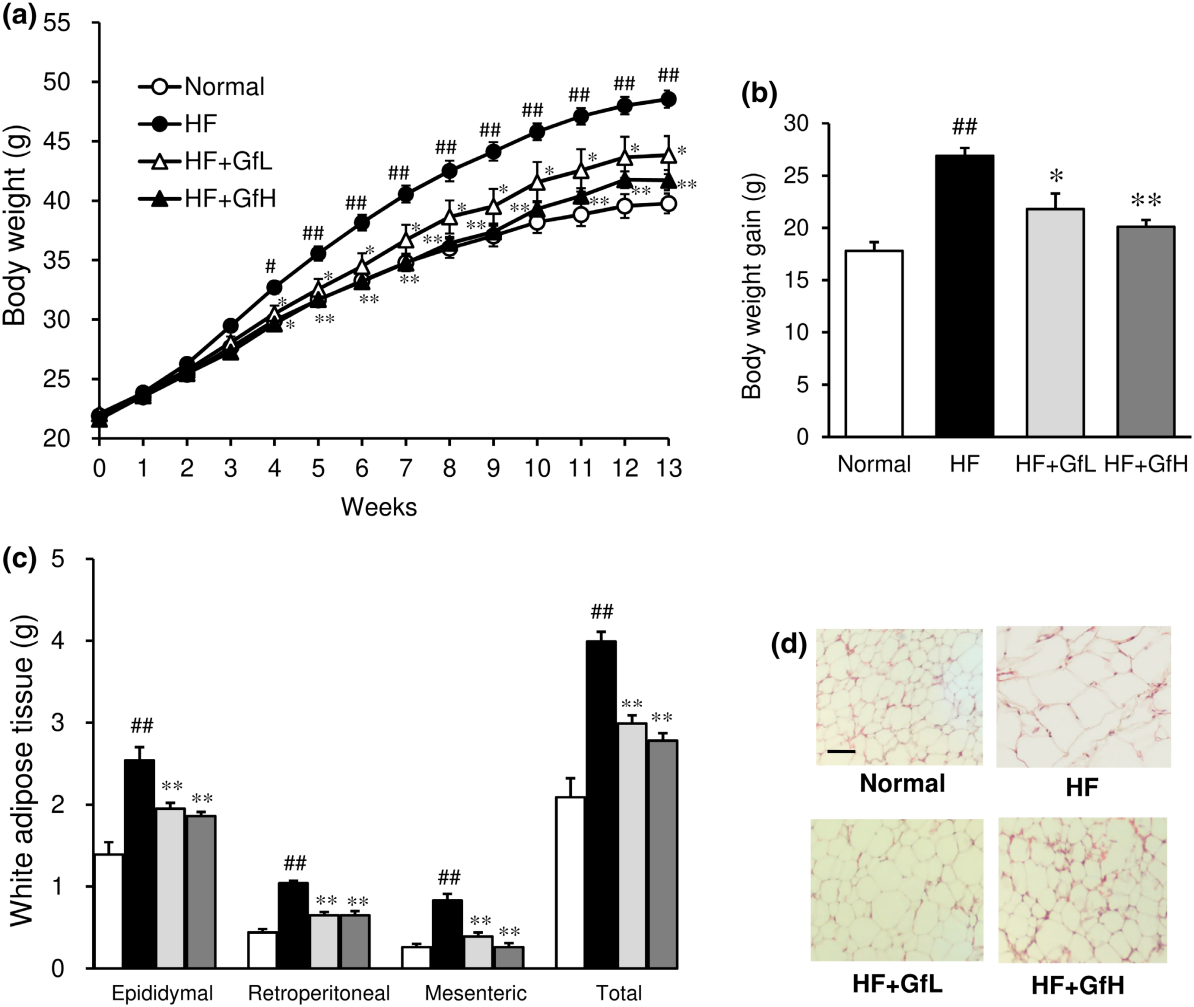
Effects of *Gloiopeltis furcata* on weekly changes in body weight (a), body weight gain (b), mass (c), and morphology (d) of white adipose tissue in C57BL/6J mice fed a high‐fat diet. Normal, normal diet; HF, high‐fat diet; HF+GfL, high‐fat diet mixed with 2% *G. furcata*; HF+GfH, high‐fat diet mixed with 6% *G. furcata*. Each value represents the mean ± *SEM* (*n* = 8–13). Statistical difference: *p < 0.05, **p < 0.01 vs. HF group, #*p* < 0.05, ##*p* < 0.01 vs. Normal group. Scale bar = 100 μm.

HF mice showed a significant increase in body weight from 4 weeks after the initiation of HF diet feeding, in comparison to normal mice (Figure 1a). The body weights of HF mice and mice fed an HF diet supplemented with *G. furcata* began to differ significantly after 4 weeks of treatment. The body weight gain was also significantly suppressed by both doses of *G. furcata* (Figure 1b). The daily food intake of each group throughout the experiment period was 3.0 g (Normal), 2.4 g (HF), 2.5 g (HF+GfL), and 2.5 g (HF+GfH), and there is no significant difference between the HF diet group and the *G. furcata*‐treated group.

### 3.3 Morphology and the white adipose tissue mass

The white adipose tissue weight, including epididymal, retroperitoneal, and mesenteric adipose tissues, was significantly increased by HF diet feeding for 13 weeks (Figure 1c). The total weight of white adipose tissue in the HF diet group was double that of the normal group. The treatment of HF mice with *G. furcata* reduced the white adipose tissue weight in each part and the total weight. Although adipocyte hypertrophy was seen in the HF diet group in comparison to the normal group, treatment with *G. furcata* suppressed the enlargement of adipocytes (Figure 1d).

### 3.4 Serum levels of glucose and insulin, and insulin resistance

**FIGURE 2:**
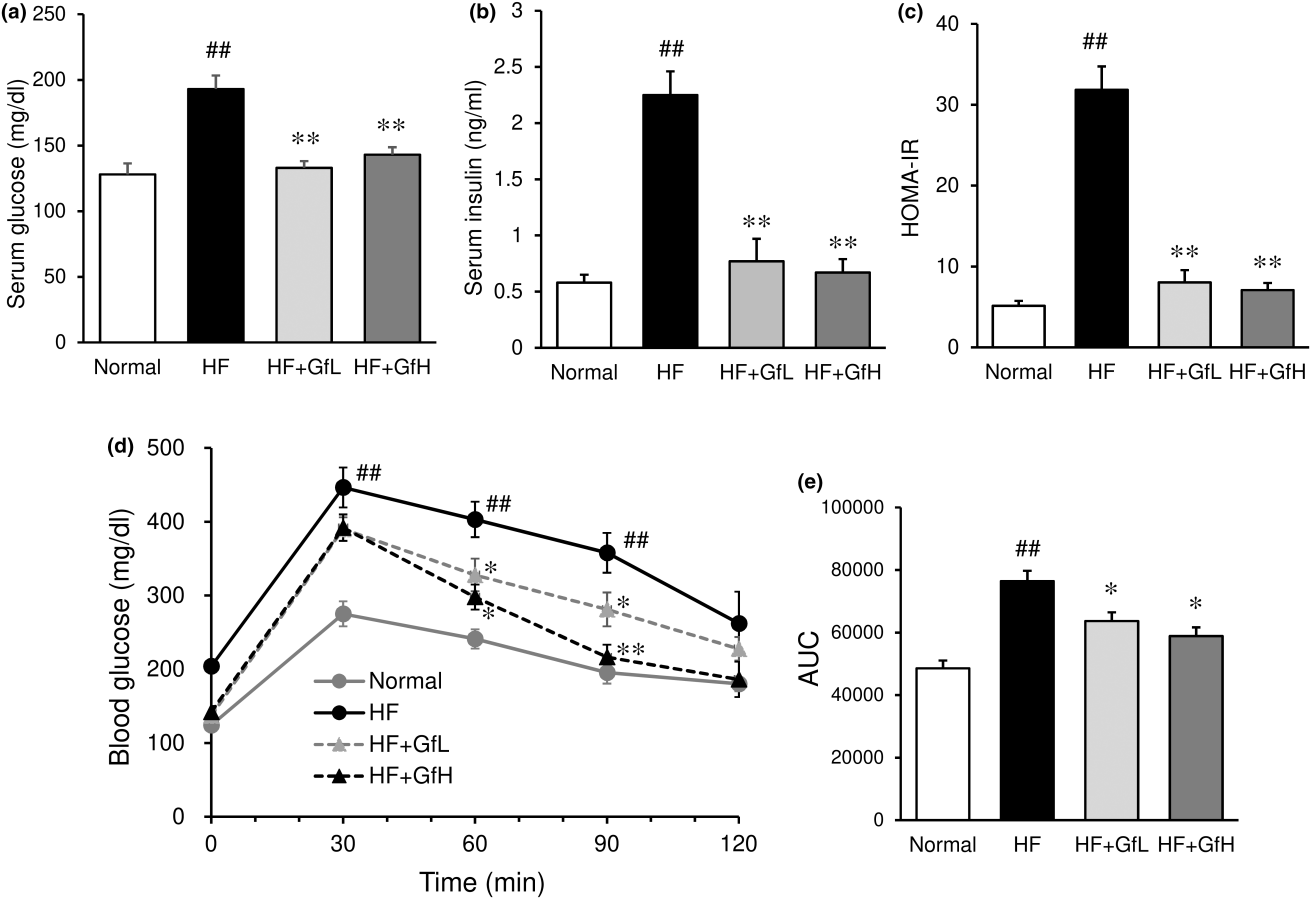
Effects of *Gloiopeltis furcata* on the serum glucose (a), insulin levels (b), HOMA‐IR (c), and insulin resistance (d) in C57BL/6J mice fed a high‐fat diet. The area under the curve (AUC) was also calculated (e). Normal, normal diet; HF, high‐fat diet; HF+GfL: high‐fat diet mixed with 2% *G. furcata*; HF+GfH: high‐fat diet mixed with 6% *G. furcata*. Each value represents the mean ± *SEM* (*n* = 6–8). Statistical difference: *p < 0.05, **p < 0.01 vs. HF group, ##*p* < 0.01 vs. Normal group.

Fasting serum levels of glucose and insulin were significantly elevated by an HF diet (Figure 2a,b). Supplementation of HF mice with *G. furcata* markedly suppressed the elevation of glucose and insulin levels (Figure 2a,b). The HOMA‐IR value, which is an index of insulin resistance, was markedly increased by ingestion of an HF diet, but was ameliorated by *G. furcata* supplementation (Figure 2c). Increased insulin resistance due to HF diet feeding was significantly suppressed in HF mice supplemented with *G. furcata* (Figure 2d). The blood glucose levels in the *G. furcata*‐treated group were significantly lower at 60 and 90 min after the intraperitoneal injection of glucose in comparison to those in the HF diet group. The area under the curve (AUC) for glucose in *G. furcata*‐treated mice was also significantly lower in comparison to HF mice (Figure 2e).

### 3.5 Serum levels of adipokines and oxidative stress markers

**FIGURE 3:**
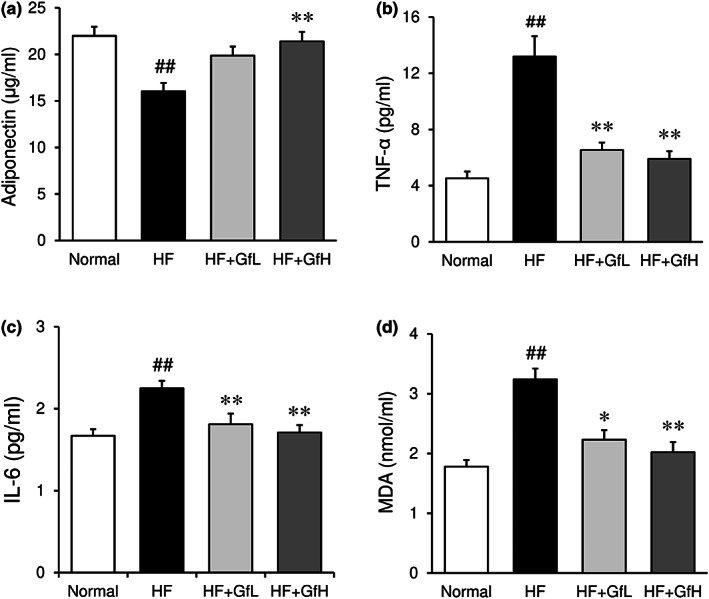
Effects of *Gloiopeltis furcata* on serum levels of adipokines and malondialdehyde (MDA) in C57BL/6J mice fed a high‐fat diet. Serum levels of adiponectin (a), TNF‐α (b), IL‐6 (c), and MDA levels (d) were determined. Normal, normal diet; HF, high‐fat diet; HF+GfL, high‐fat diet mixed with 2% *G. furcata*; HF+GfH, high‐fat diet mixed with 6% *G furcata*. Each value represents the mean ± *SEM* (*n* = 6–8). Statistical difference: *p < 0.05, **p < 0.01 vs. HF group, ##*p* < 0.01 vs. Normal group.

Serum levels of anti‐inflammatory adiponectin were significantly reduced by an HF diet (Figure 3a), while proinflammatory adipokines tumor necrosis factor‐α (TNF‐α) and interleukin‐6 (IL‐6) levels were increased by an HF diet (Figure 3b,c). Supplementation of HF mice with *G. furcata* normalized these changes. In addition, the serum level of malondialdehyde (MDA), a marker of lipid peroxidation, was elevated by an HF diet, which was also suppressed by supplementation with *G. furcata* (Figure 3d).

### 3.6 Hepatic steatosis and liver damage markers

**FIGURE 4:**
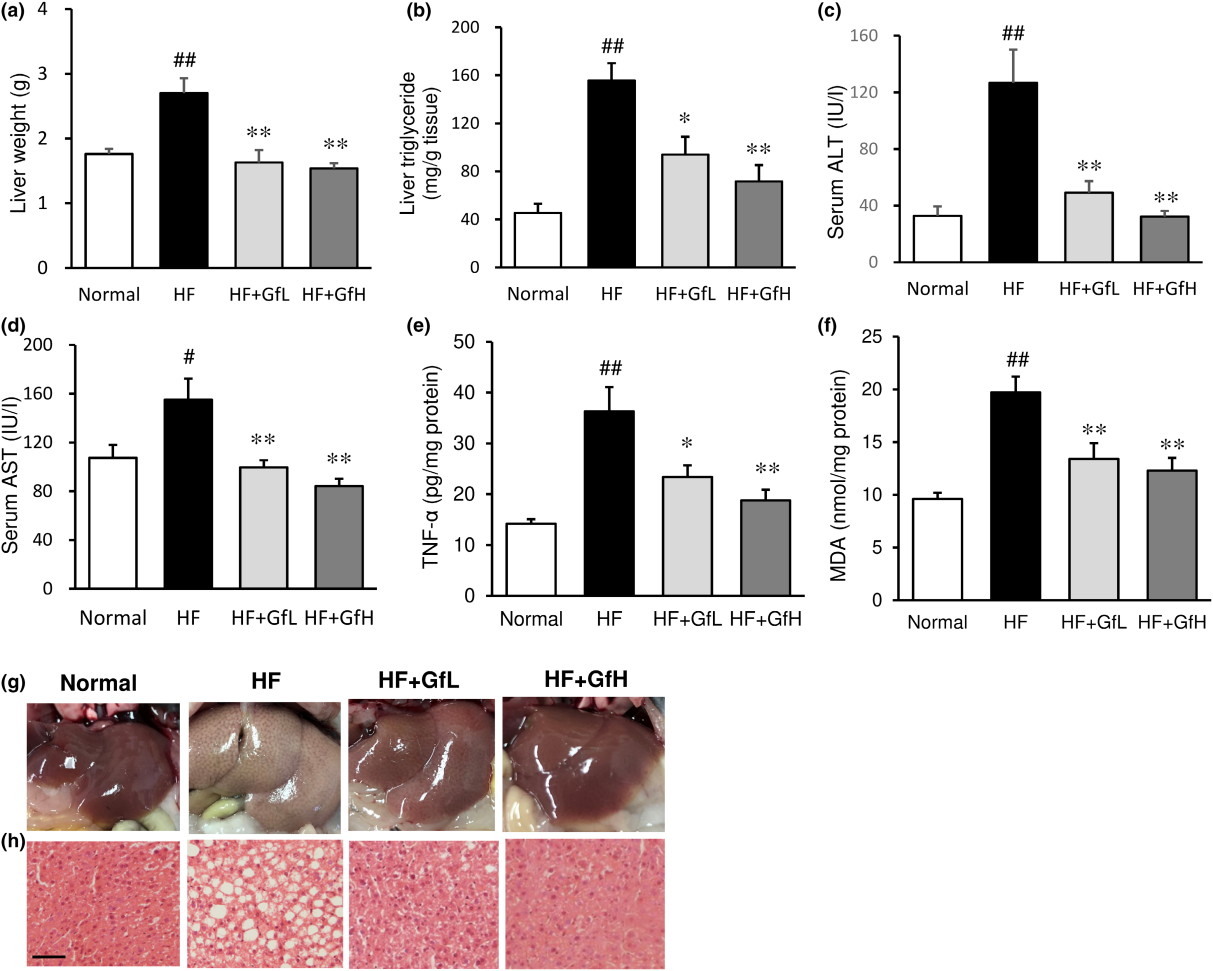
Effects of *Gloiopeltis furcata* on the liver weight, hepatic lipid accumulation, and serum parameters of the liver function in C57BL/6J mice fed a high‐fat diet. (a) Liver weight; (b) Liver triglyceride content; Serum levels of (c) alanine aminotransferase (ALT) and (d) aspartate aminotransferase (AST); Hepatic levels of (e) TNF‐α and (f) MDA; (g) Representative gross morphology and (h) histological sections of the liver. Normal, normal diet; HF, high‐fat diet; HF+GfL, high‐fat diet mixed with 2% *G. furcata*; HF+GfH, high‐fat diet mixed with 6% *G. furcata*. Each value represents the mean ± *SEM* (*n* = 6–8). Statistical difference: *p < 0.05, **p < 0.01 vs. HF group, #*p* < 0.05, ##*p* < 0.01 vs. Normal group. Scale bar = 100 μm.

The ingestion of an HF diet for 13 weeks increased the liver triglyceride content more than threefold; this was accompanied by a significant increase in the liver weight (Figure 4a,b). Macroscopic observation showed that the livers of mice in the HF diet group were enlarged in comparison to the normal group and exhibited a whitish color (Figure 4g). A histological examination revealed that ingestion of HF diet caused hepatic steatosis, as was evident from changes in hepatic tissue, including the occurrence of vacuoles, lipid droplets, and hepatocyte swelling (Figure 4h). Treatment of HF mice with *G. furcata* suppressed the increase in liver weight and liver triglyceride content (Figure 4a,b). It also ameliorated the HF diet‐induced pathological changes (Figure 4g,h). Consistent with the reduced liver triglyceride content and improved histology, HF diet‐induced elevation of serum parameters of the liver function, including alanine aminotransferase (ALT) and aspartate aminotransferase (AST) was markedly suppressed by treatment with *G. furcata* (Figure 4c,d).

### 3.7 Hepatic levels of adipokines and oxidative stress marker

The ingestion of an HF diet significantly increased the hepatic levels of proinflammatory TNF‐α and a marker of lipid peroxidation, MDA (Figure 4e,f). Treatment with *G. furcata* suppressed the elevation of these parameters.

### 3.8 Serum lipid levels

**FIGURE 5:**
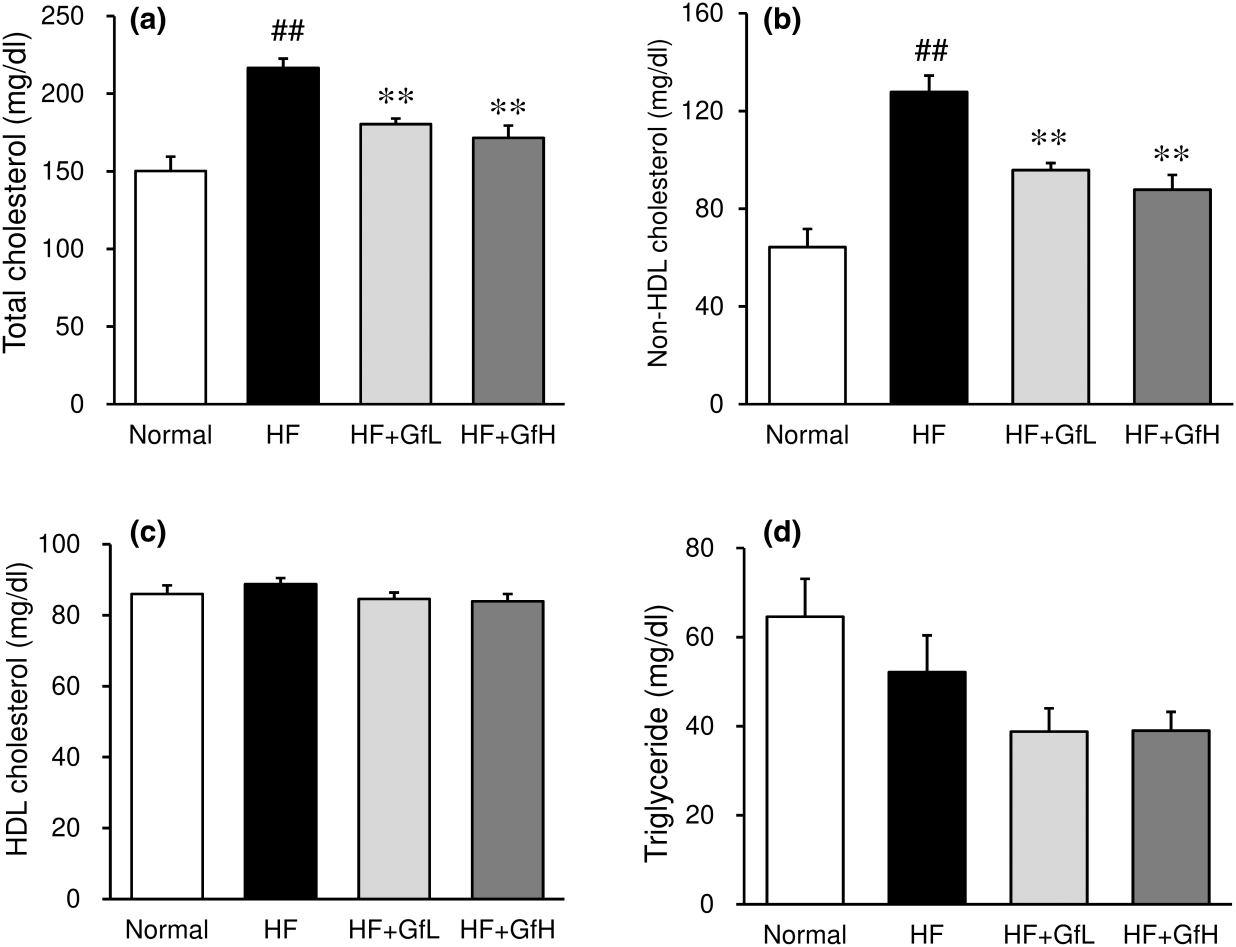
Effects of *Gloiopeltis furcata* on serum lipid levels in C57BL/6J mice fed a high‐fat diet. Serum levels of total cholesterol (a), HDL‐cholesterol (c), and triglyceride (d) were determined. Non‐HDL cholesterol (b) was calculated by subtracting HDL‐cholesterol from total cholesterol. Normal, normal diet; HF, high‐fat diet; HF+GfL, high‐fat diet mixed with 2% *G. furcata*; HF+GfH, high‐fat diet mixed with 6% *G. furcata*. Each value represents the mean ± *SEM* (*n* = 8). Statistical difference: **p < 0.01 vs. HF group, ##*p* < 0.01 vs. Normal group.

The serum levels of total cholesterol and non‐HDL cholesterol were significantly increased in the HF diet group in comparison to those in the normal group (Figure 5a,b). Treatment with *G. furcata* suppressed the elevation of these lipid levels in a dose‐dependent manner. In contrast, there were no significant changes in serum levels of HDL cholesterol and triglyceride, which were not affected by the treatment with *G. furcata* (Figure 5c,d).

### 3.9 Fecal weight and fecal content of triglyceride and polysaccharides

**FIGURE 6:**
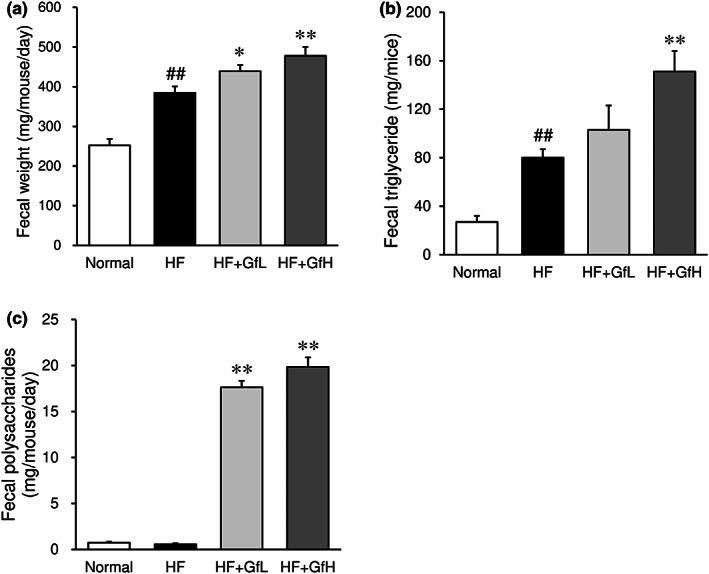
Effects of *Gloiopeltis furcata* on the fecal weight and feces components in C57BL/6J mice fed a high‐fat diet. (a) Fecal weight; (b) Fecal triglyceride; (c) Fecal polysaccharides. Normal, normal diet; HF, high‐fat diet; HF+GfL, high‐fat diet mixed with 2% *G. furcata*; HF+GfH, high‐fat diet mixed with 6% *G. furcata*. Each value represents the mean ± *SEM* (*n* = 7). Statistical difference: *p < 0.05, **p < 0.01 vs. HF group, ##*p* < 0.01 vs. Normal group.

The ingestion of an HF diet significantly increased the fecal weight, and treatment with *G. furcata* further increased the fecal weight (Figure 6a). Similarly, the increase in fecal triglyceride induced by the HF diet was further increased by treatment with *G. furcata* (Figure 6b). The fecal polysaccharide content in the *G. furcata*‐treated group was markedly increased in comparison to the HF diet and normal groups (Figure 6c).

### 3.10 Production of nitric oxide and TNF‐α, and pancreatic lipase activity

**FIGURE 7:**
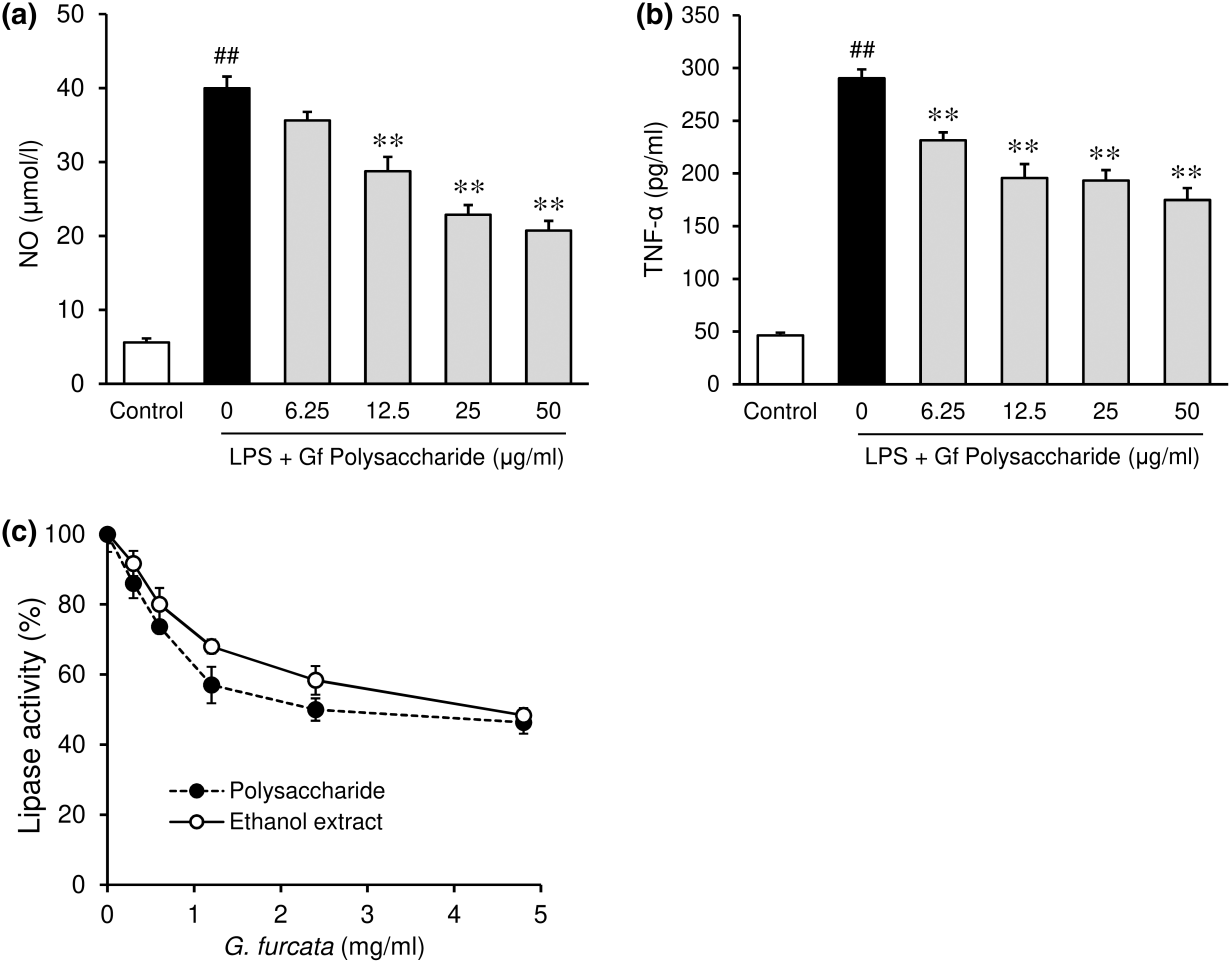
Effects of polysaccharide and ethanol extract from *Gloiopeltis furcata* on the production of nitric oxide (a) and TNF‐α (b), and the pancreatic lipase activity (c). Polysaccharide was purified from *G. furcata* (Gf), and its effects on the production of nitric oxide (NO) and TNF‐α were assessed in RAW 264.7 cells. Each value represents the mean ± *SEM* (*n* = 6). Statistical difference: **p < 0.01 vs. LPS Gf polysaccharide (0 μg/ml), ##*p* < 0.01 vs. Control. Effects of polysaccharides and ethanol extract from *G. furcata* on the pancreatic lipase activity were determined in vitro (c). Each point represents the mean ± *SEM* of triplicate experiments.

Anti‐inflammatory activity of purified polysaccharide from *G. furcata* was evaluated in lipopolysaccharide (LPS)‐treated RAW264.7 cells. The marked increase in nitric oxide (NO) and TNF‐α production by LPS treatment was suppressed by increasing concentrations of polysaccharide (Figure 7a,b). The inhibitory effect on the pancreatic lipase activity was assessed in vitro. Extracts of 70% ethanol and polysaccharide from *G. furcata* inhibited the lipase activity in a dose‐dependent manner (Figure 7c).

## 4 DISCUSSION

We investigated the effects of an edible red seaweed, *Gloiopeltis furcata* (Postels et Ruprecht) J. Agardh (*G. furcata*) on diet‐induced obesity in mice. Thirteen‐week treatment of high‐fat (HF) mice with two doses of *G. furcata* attenuated the development of obesity, as was evident from the suppression of the increase in the body weight and white adipose tissue weight. In addition, dietary *G. furcata* attenuated obesity‐related metabolic disorders, including insulin resistance, hyperglycemia, hepatic steatosis, and dyslipidemia. These results suggest that Japanese traditional seaweed *G. furcata* may be a beneficial ingredient for the treatment of obesity‐ and diabetes‐related metabolic disorders.

Triglycerides represent the main constituents of dietary fat. Pancreatic lipase is a key enzyme that catalyzes the hydrolysis of triglycerides to monoacylglycerols and fatty acids. The resulting fatty acids are absorbed from the brush border of the small intestine. Therefore, pancreatic lipase is targeted for the development of anti‐obesity agents (Wan‐Loy & Siew‐Moi, 2016). Thus far, many natural products with diverse structures, including polyphenols and polysaccharides have been reported to inhibit pancreatic lipase (Buchholz & Melzig, 2015; Lunagariya et al., 2014). A fecal analysis showed that the ingestion of *G. furcata* increased the fecal triglyceride content, which was accompanied by an increase in fecal polysaccharides content. This finding indicates that the inhibition of intestinal absorption and the subsequent stimulation of fecal excretion of dietary lipids are the major mechanisms by which *G. furcata* prevented obesity and related metabolic disorders. Seaweed is rich in polysaccharide, which is known to exhibit a wide variety of biological activities, including antiviral, antibacterial, anticoagulant, anti‐inflammatory, antioxidant, hypolipidemic, and hypotensive effects (Hentati et al., 2020; Xu et al., 2017). To elucidate the major components responsible for the anti‐obesity and anti‐diabetic effects, we isolated polysaccharide from *G. furcata*. An analysis of polysaccharide using high‐performance size‐exclusion chromatography revealed that the average molecular weight of the polysaccharide was 1.8 × 10^6^ Da, with sulfate content similar to that of κ‐carrageenan, and its solution exhibited high viscosity. An in vitro study showed that purified polysaccharide inhibited the pancreatic lipase activity in a dose‐dependent manner. Thus, high‐viscosity sulfated polysaccharide is a major component of *G. furcata* responsible for the inhibition of lipase activity and intestinal lipid absorption. Some seaweed polysaccharides have been shown to inhibit the pancreatic lipase activity (Austin et al., 2018; Wilcox et al., 2014). As our analysis showed that the 70% ethanol extract contained polyphenols, and *G. furcata* has been reported to contain rich phenolic compounds, such as phlorotannins (Yang et al., 2010), phenolic compounds may also be partly involved in the inhibition of pancreatic lipase. Phenolic derivatives have been shown to ameliorate obesity via several mechanisms, including the inhibition of pancreatic lipase (Wan‐Loy & Siew‐Moi, 2016).

The polysaccharides are not digested by the intestinal enzymes and act as soluble fibers in the gastrointestinal tract. The soluble fibers have been shown to interfere with intestinal lipid absorption by several mechanisms other than the inhibition of pancreatic lipase. For example, they are dispersible in water and become viscous, which affects the rate of gastric emptying and results in slower rates of digestion and absorption of intestinal contents (Lin et al., 1997). The soluble fibers are also known to suppress appetite and energy intake in the digestive tract through several physiological mechanisms, which include the effects of physical structure, the water‐holding capacity, and viscosity (Kristensen & Jensen, 2011). Considering the high viscous property of polysaccharide from *G. furcata*, the physicochemical characteristics may also be associated with the decreased intestinal lipid absorption.

Recently, many soluble fermentable fibers, including those from seaweed, have demonstrated a prebiotic effect and alter the intestinal microflora composition toward a more beneficial distribution (de Jesus, Raposo, et al., 2016; Sanders et al., 2019). These characteristics of polysaccharides are associated with the reduced absorption of triglyceride and glucose, and may be partly responsible for the amelioration of obesity and related metabolic disorders (Bouter et al., 2017). Furthermore, soluble fibers interact with bile acids and increase their fecal excretion (Story & Furumoto, 1990), which is thought to contribute to the cholesterol‐lowering effect of *G. furcata* in the present study.

The ingestion of *G. furcata* attenuated the accumulation of body fat, which was accompanied by the suppression of adipocyte hypertrophy. Adipose tissue is now recognized as an endocrine organ, capable of secreting a large number of bioactive factors, known as adipokines, which regulate a wide variety of physiological functions (Fasshauer & Blüher, 2015). It is well established that obesity represents a state of low‐grade chronic inflammation linked to metabolic disorders, such as insulin resistance and type 2 diabetes (Hotamisligil, 2006). Adiponectin is among the adipokines that exert a beneficial impact on obesity, insulin resistance, and cardiovascular disease and also exert anti‐inflammatory effects (Fang & Judd, 2018). Clinical and experimental observations indicate that low plasma levels of adiponectin contribute to the pathogenesis of insulin resistance and type 2 diabetes in obese patients and obese animals (Frankenberg et al., 2017). In contrast, TNF‐α is a key pro‐inflammatory adipokine secreted by adipocytes. The increased level of TNF‐α induces insulin resistance in adipocytes and peripheral tissues by impairing the insulin signaling, which leads to the development of type 2 diabetes (Moller, 2000). With the development of lipid accumulation in adipose tissue, the pattern of adipokine secretion by adipocytes changes with a decrease in adiponectin and an increase in TNF‐α. Consistent with these previous observations, the ingestion of an HF diet by mice decreased their serum adiponectin levels and markedly increased their serum TNF‐α levels in the present study. The changes in serum adipokine levels were associated with the development of obesity, insulin resistance, and the elevation of serum glucose levels, as well as hypertrophic changes in adipocytes. Supplementation with *G. furcata* suppressed the hypertrophy of adipocytes and ameliorated the development of obesity, diabetes, hepatic steatosis, and dyslipidemia, which was accompanied by a dose‐dependent recovery of serum adiponectin and TNF‐α to normal levels. These results suggest that the anti‐inflammatory effect on adipose tissue may be partly associated with the amelioration of obesity by *G. furcata*. The present study showed that polysaccharide from *G. furcata* suppressed the lipopolysaccharide (LPS)‐stimulated production of nitric oxide (NO) and TNF‐α in RAW 264.7 cells. Whether or not polysaccharide from *G. furcata* is absorbed into the body is unclear. However, several studies have shown that fucoidan, a sulfated polysaccharide from brown seaweed, is absorbed and distributed in the body in rats (Bai et al., 2020) and humans (Kadena et al., 2018). Ethyl acetate extract of *G. furcata* has been reported to inhibit the LPS‐stimulated production of pro‐inflammatory substances, including NO, PGE2, IL‐6, and TNF‐α in murine macrophage RAW 264.7 cells (Yang et al., 2010). Thus, polysaccharides and polyphenols may be major active components involved in the anti‐inflammatory activity of *G. furcata*, as *G. furcata* is also high in phlorotannin content (Yang et al., 2010).

It is also established that reactive oxygen species (ROS) or oxidative stress are closely involved in the onset and development of obesity and related metabolic disorders, in addition to inflammation (Le Lay et al., 2014). Elevated levels of oxidative stress markers, such as MDA or thiobarbituric acid reactive substances (TBARS) have been observed in obese experimental animals and obese individuals (Degasperi et al., 2009; Zhang et al., 2015). In mice with HF diet‐induced obesity, elevated mitochondrial ROS formation has been shown to contribute to the induction of insulin resistance in white adipose tissue (Paglialunga et al., 2015). Furthermore, proinflammatory adipokines, including TNF‐α and IL‐6 are known to stimulate the production of ROS by macrophages and monocytes (Fernández‐Sánchez et al., 2011), indicating that elevated levels of proinflammatory adipokines can be responsible for increased oxidative stress in an obese state. An HF diet has been demonstrated to be a significant risk factor for health. Animals subjected to long‐term HF diet feeding show increased oxidative stress and dysfunctional mitochondria in several organs (Ballal et al., 2010). Actually, the treatment of HF mice with *G. furcata* reduced elevated levels of MDA, as a marker of oxidative stress, in both the liver and serum. This finding suggests that suppression of oxidative stress is also a possible mechanism by which *G. furcata* attenuated the development of obesity and related‐metabolic disorders. Fucoidan, a sulfated polysaccharide derived from brown seaweed, has been shown to attenuate insulin resistance and hepatic steatosis by suppressing oxidative stress and inflammation in rats fed an HF diet (Heeba & Morsy, 2015). Antioxidative phenolic compounds, such as phlorotannins from marine sources, have been reported to exhibit anti‐obesity and anti‐diabetic effects in animal models (Mateos et al., 2020). We have previously reported that the anti‐obesity effects of brown and red seaweed consumed in Japan are related to their antioxidant and anti‐inflammatory properties (Murakami et al., 2021, 2022).

## 5 CONCLUSIONS

In summary, our current study demonstrated that supplementation with *G. furcata* ameliorated the development of obesity, diabetes, and related metabolic disorders in mice fed an HF diet. The beneficial effect of *G. furcata* was associated with the inhibition of intestinal lipid absorption, and the suppression of inflammation and oxidative stress. Polysaccharide and polyphenols play important roles in the amelioration of these metabolic disorders by *G. furcata*. Thus, *G. furcata* may be useful for preventing obesity, diabetes, hepatic steatosis, and hypercholesterolemia, although clinical investigations are necessary to confirm its effectiveness in humans.

## CONFLICT OF INTEREST

The authors declare no conflict of interest.

## ETHICAL APPROVAL

All methods were carried out in accordance with relevant guidelines and regulations. All experimental protocols for animals were approved by the Institutional Animal Care and Use Committee of Fukui Prefectural University (approval No. 19‐14). The animal care and experimental procedures were carried out in compliance with the ARRIVE guidelines.

## Data Availability

The data that support the findings of this study are available from the corresponding author upon reasonable request.
